# Supplementary material for: Homo- and hetero-dimeric subunit interactions set affinity and efficacy in metabotropic glutamate receptors
Source: Nat Commun. 2023 Dec 13;14:8288. doi: 10.1038/s41467-023-44013-4 (PMC10719366; doi:10.1038/s41467-023-44013-4)

Homo- and hetero-dimeric subunit interactions set affinity and efficacy in metabotropic glutamate receptors

Chris Habrian, Naomi Latorraca, Zhu Fu and Ehud Y. Isacoff

Supplemental Material

Supplemental Figures

Supplemental Figure 1. Single molecule FRET assay.

(a) Schematic of individual dimer antibody pulldown and immobilization on PEG passivated coverslip for TIRF single molecule imaging of donor and acceptor dyes.

(**b**) Single frame of movie recording donor and acceptor fluorescence channels.

(**c**) Individual traces of SNAP-mGluR2 in 0 glutamate (top) and 1mM glutamate (bottom). Green trace is donor fluorescence, red trace is acceptor fluorescence and blue trace is FRET trace as calculated by acceptor fluorescence divided by total (donor + acceptor) fluorescence. Photobleaching of the acceptor at ~45 s for the 0 glutamate trace and ~51 s for the 1 mM glutamate trace de-quenches and increases brightness of the donor because of termination of energy transfer following acceptor destruction. All points histogram of FRET levels from the shown dimer trace on right. Compiled histograms from many dimers in 0 glutamate and 1 mM glutamate shown on right with red dashed lines showing histogram centers.

(**d**) Schematic of N-terminally labeled mGluR with donor (green) on one subunit and acceptor (red) on the other in resting/open-open state (top) and active/closed-closed state (bottom).

Supplemental Figure 2. LBD models of ligand binding pocket and dimer interfaces.

(a) Residue conservation across a subset (n=8) of mGluR4 and mGluR7 sequences. Backbone coded from high (blue) to low (red) conservation. Ligand interacting residues shown as stick figures.

(b) Dimer interfaces between LBD upper lobes. LBD residues 41-518 shown. Colors show Q1 (residues 40-201, purple) and Q2 (residues 202-340, teal).

Structures are of the mGluR4/4 homodimer in the active state (PDB 7E9H).

Supplemental Figure 3. Subregions of mGluR4 LBD lower lobe interface region 202-271 do not increase mGluR7 ligand efficacy.

(**a**) Sequence alignment of mGluR4 and mGluR7 of lower interface residues 202-271 displaying three subregions: Section 1, Section 2 and Section3 .

(**b**) SNAP-mGluR7(Section 1+2).

(**c**) SNAP-mGluR7(Section 1+3).

(**d**) SNAP-mGluR7(Section 2+3).

Values shown as mean $\pm$ s.e.m.

Supplemental Figure 4. Sequence alignment of mGluR4 and mGluR7.

Sequence alignment of mGluR4 and mGluR7 highlighting amino acid changes made within the cysteine loop.

Supp. Figure 1

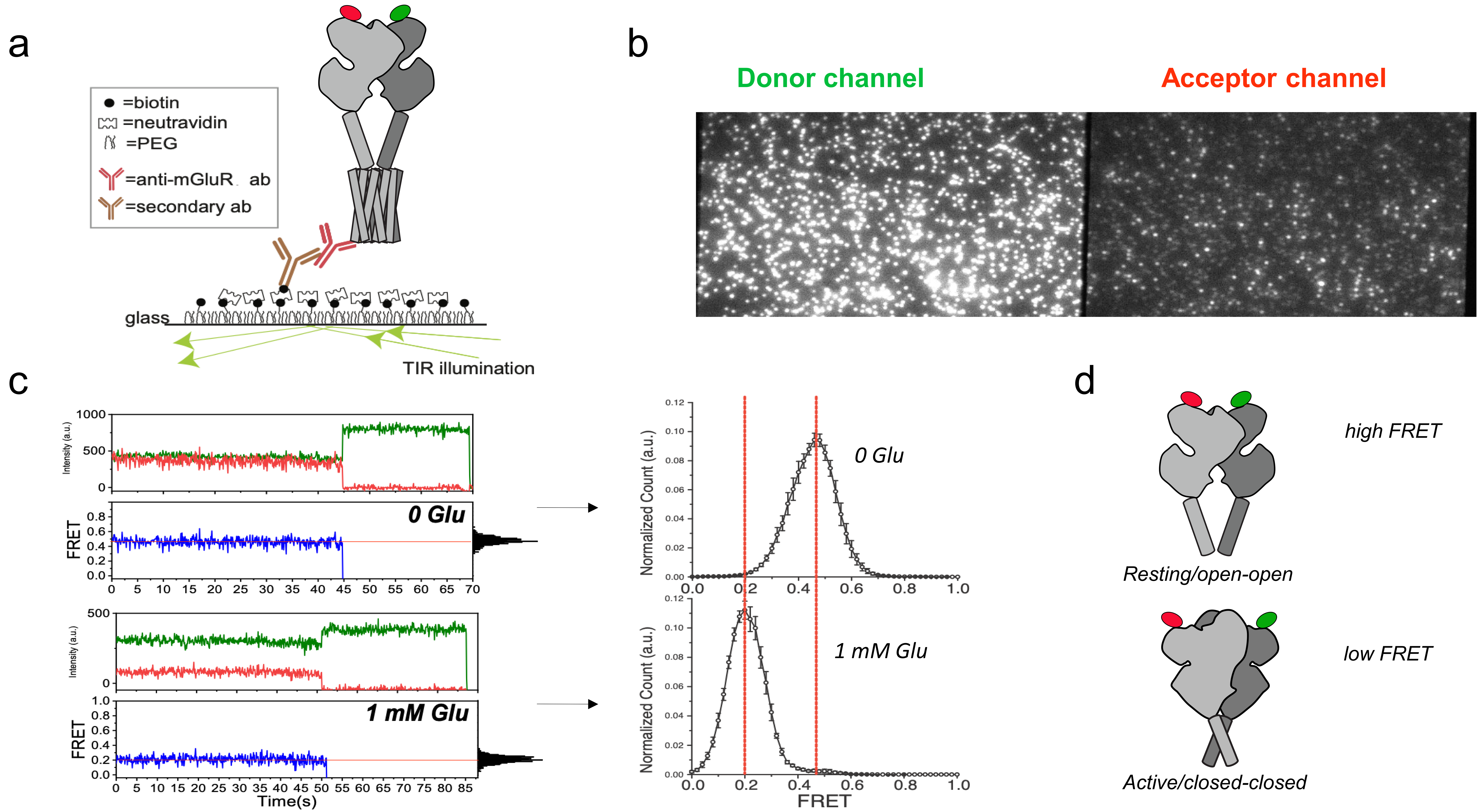

Habrian Suppl. Figure 2

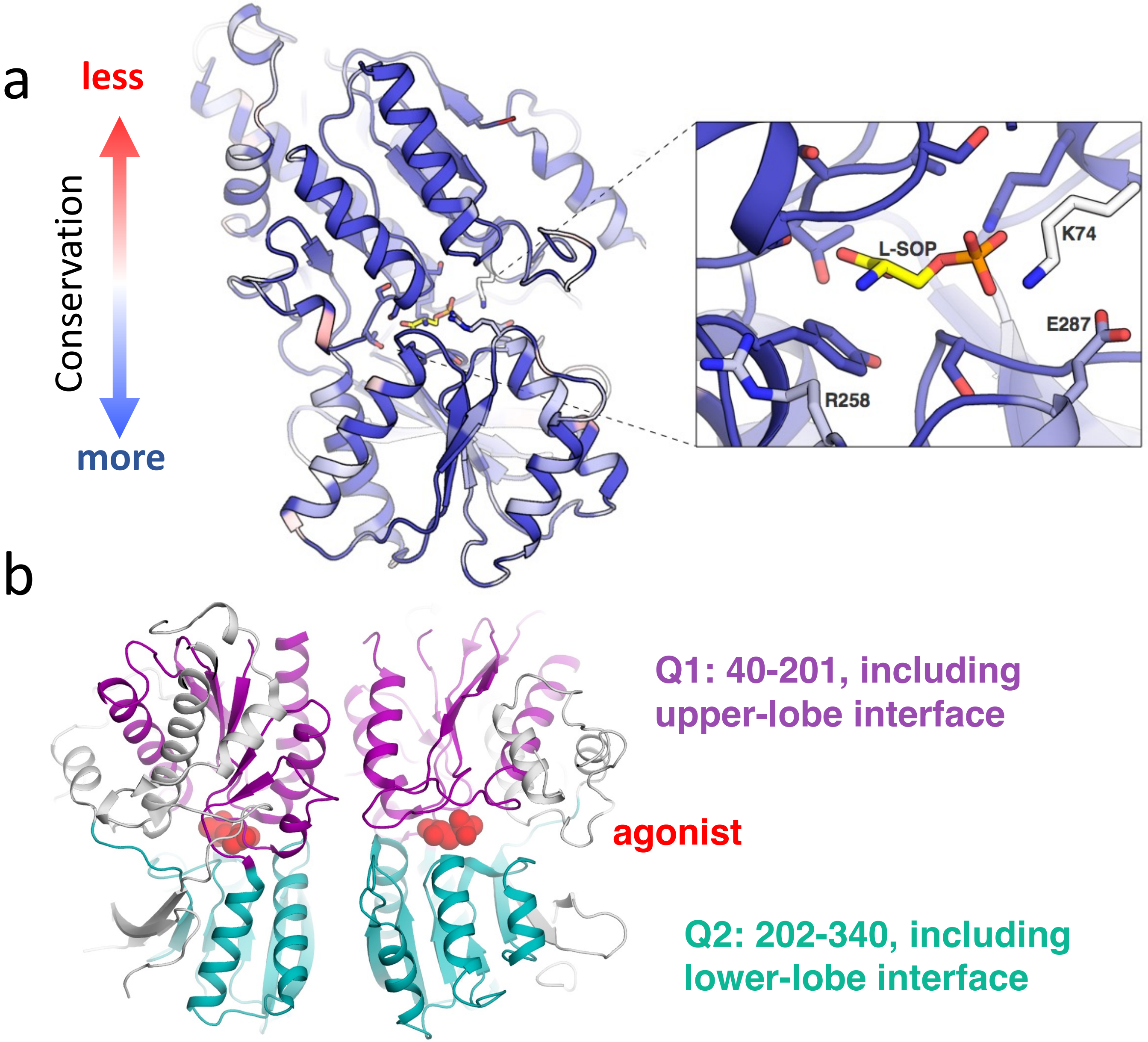

Habrian Suppl. Figure 3

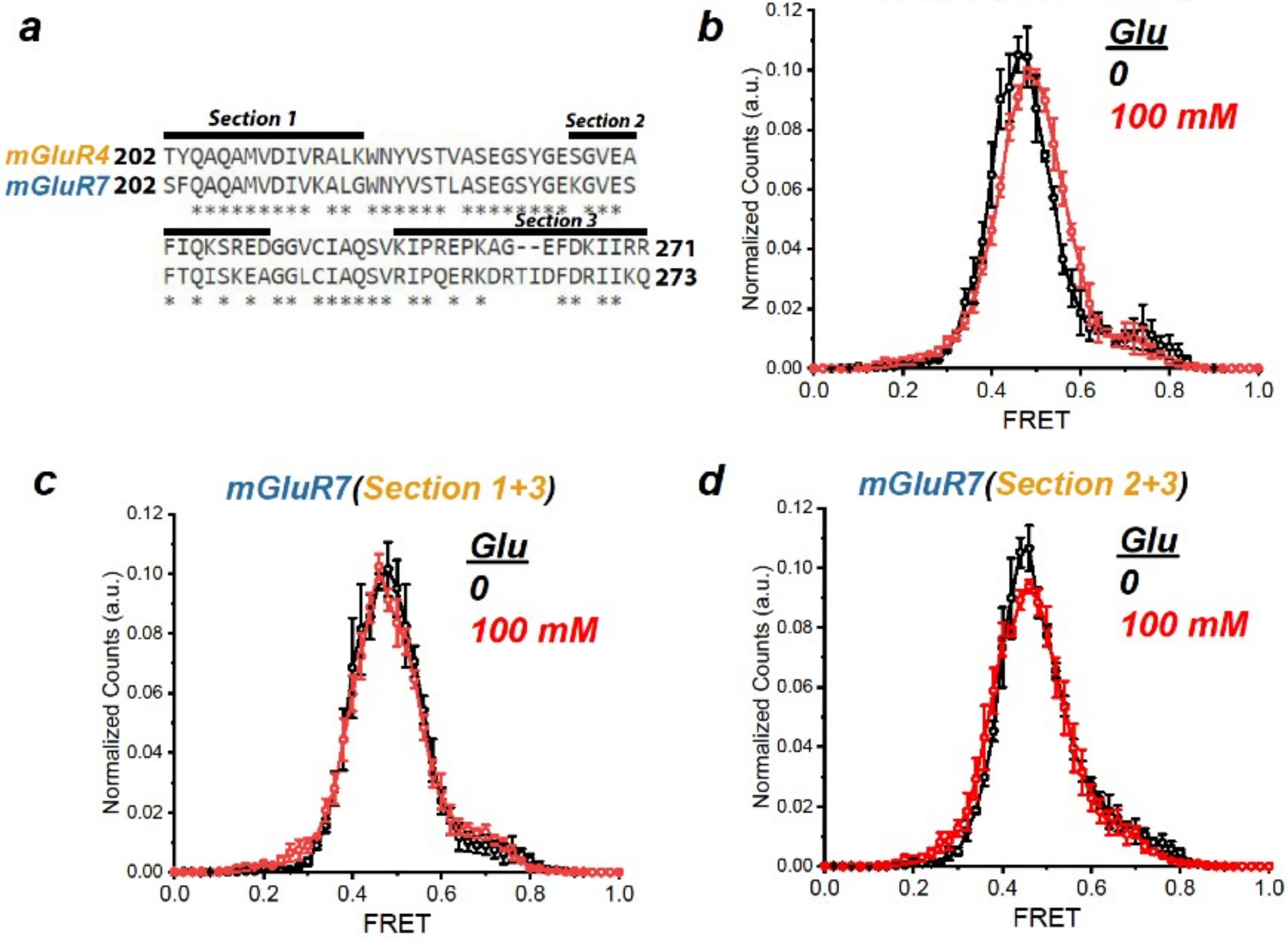

Habrian Suppl. Figure 4

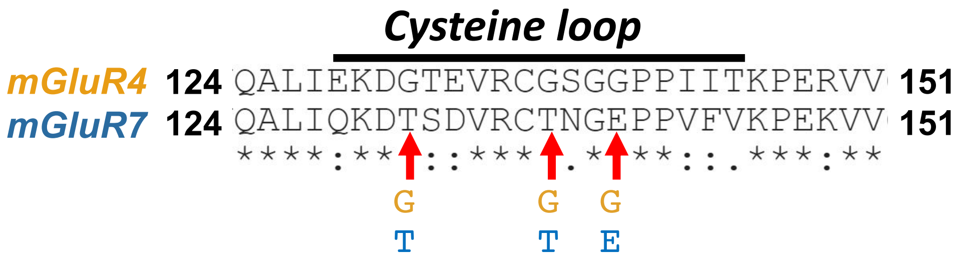

Supplement: Supplementary file 1 — Supplementary Information [file 41467_2023_44013_MOESM1_ESM.pdf]
